# Supplementary material for: The Effects of Mindfulness Meditation on Burnout in Clinical Genetic Counselors: A Three-Arm Randomized Controlled Trial
Source: medRxiv. 2026 Jan 16:2026.01.14.26344130. Preprint. [Version 1] doi: 10.64898/2026.01.14.26344130 (PMC12870655; doi:10.64898/2026.01.14.26344130)
Supplement: Supplement 1 [file NIHPP2026.01.14.26344130v1-supplement-1.pdf]

**Supplemental Table 1. Properties and timing of scales used**

| Construct(s) measured | Scale | Data collection schedule | | | Scale properties | | |
|---|---|---|---|---|---|---|---|
| | | T0 | T1 | T2[a] | Internal consistency (Cronbach's α) | Test-re-test reliability (r) | Items |
| Mindfulness | FFMQ | x | x | x | 0.75-0.91 | unavailable | 39 |
| Credibility & Expectancy | CEQ[b] | x | | | 0.84-0.85 | 0.83 | 6 |
| Burnout & Professional fulfillment | PFI | x | x | x | 0.80-0.92 | 0.71-0.82 | 16 |
| Stress | PSS | x | x | x | 0.91 | 0.75-0.77 | 10 |
| Self-care behaviors | PSCS | x | x | x | 0.70-0.83 | unavailable | 21 |
| Resilience | CD-RISC | x | x | x | 0.85 | unavailable | 10 |
| Reactive distress | IRI subscale | x | x | x | 0.73-0.79 | 0.61-0.81 | 14 |

[a] T2 survey was only sent to MM participants. [b] Measured only in MM and ACM participants and administered 1 week into the intervention period. FFMQ = Five-Factor Mindfulness Questionnaire. CEQ = Credibility and Expectancy Questionnaire. PFI = Professional Fulfillment Inventory. PSS = Perceived Stress Scale. PSCS = Professional Self-Care Scale. CD-RISC = Connor-Davidson Resilience Scale.

medRxiv preprint doi: https://doi.org/10.64898/2026.01.14.26344130; this version posted January 16, 2026. The copyright holder has placed this preprint (which was not certified by peer review) in the Public Domain. It is no longer restricted by copyright. Anyone can legally share, reuse, remix, or adapt this material for any purpose without crediting the original authors.

**Supplemental Table 2. Cronbach's Alpha (α) for Scales by Study Arm**

| Scale | Full Cohort (N) | ACM (N) | NMC (N) | MM (N) |
|---|---|---|---|---|
| **FFMQ** | | | | |
| Observing | 0.79 (394) | 0.77 (132) | 0.77 (132) | 0.84 (130) |
| Describing | 0.90 (391) | 0.89 (131) | 0.90 (130) | 0.90 (130) |
| Acting with Awareness | 0.88 (390) | 0.87 (132) | 0.88 (130) | 0.90 (128) |
| Nonjudging of Inner Experience | 0.94 (391) | 0.94 (130) | 0.94 (132) | 0.95 (129) |
| Nonreactivity to Inner Experience | 0.83 (394) | 0.84 (130) | 0.81 (132) | 0.85 (132) |
| **CEQ** | | | | |
| Credibility | 0.86 (177) | 0.87 (84) | — | 0.80 (93) |
| Expectancy | 0.83 (176) | 0.83 (84) | — | 0.80 (92) |
| **PFI** | | | | |
| Work Exhaustion | 0.84 (396) | 0.80 (133) | 0.85 (132) | 0.87 (131) |
| Interpersonal Disengagement | 0.87 (393) | 0.85 (132) | 0.88 (132) | 0.88 (129) |
| Burnout (composite) | 0.89 (393) | 0.88 (132) | 0.90 (132) | 0.90 (129) |
| Professional Fulfillment | 0.88 (393) | 0.87 (131) | 0.87 (132) | 0.88 (130) |
| **PSS** | 0.87 (395) | 0.85 (131) | 0.87 (132) | 0.88 (132) |
| **PSCS** | | | | |
| Total Score | 0.87 (382) | 0.87 (129) | 0.86 (127) | 0.88 (126) |
| Professional Support | 0.81 (396) | 0.82 (132) | 0.79 (132) | 0.82 (132) |
| Professional Development | 0.68 (392) | 0.67 (132) | 0.71 (130) | 0.66 (130) |
| Life Balance | 0.84 (392) | 0.87 (133) | 0.84 (129) | 0.78 (130) |
| Cognitive Awareness | 0.69 (392) | 0.70 (131) | 0.69 (131) | 0.69 (130) |
| Daily Balance | 0.44 (395) | 0.51 (133) | 0.28 (130) | 0.53 (132) |
| **CD-RISC** | 0.83 (393) | 0.82 (131) | 0.79 (131) | 0.87 (131) |
| **IRI** | | | | |

medRxiv preprint doi: https://doi.org/10.64898/2026.01.14.26344130; this version posted January 16, 2026. The copyright holder has placed this preprint (which was not certified by peer review) in the Public Domain. It is no longer restricted by copyright. Anyone can legally share, reuse, remix, or adapt this material for any purpose without crediting the original authors.

| | | | | |
|---|---|---|---|---|
| Personal Distress | 0.80 (392) | 0.81 (132) | 0.76 (129) | 0.83 (131) |

Cronbach's α values represent internal consistency for each scale or subscale within cohort groups at T0. ACM = Active Control Mediation; NMC = No-Meditation Control; MM = Mindfulness Meditation. FFMQ = Five-Factor Mindfulness Questionnaire. CEQ = Credibility and Expectancy Questionnaire. PFI = Professional Fulfillment Inventory. PSS = Perceived Stress Scale. PSCS = Professional Self-Care Scale. CD-RISC = Connor-Davidson Resilience Scale.

medRxiv preprint doi: https://doi.org/10.64898/2026.01.14.26344130; this version posted January 16, 2026. The copyright holder has placed this preprint (which was not certified by peer review) in the Public Domain. It is no longer restricted by copyright. Anyone can legally share, reuse, remix, or adapt this material for any purpose without crediting the original authors.

**Supplemental Table 3. Prior experience with meditation and mindfulness**

| | |
|---|---|
| **Meditation training[a]** | |
| None | 141/397 (35.5%) |
| In a yoga class | 148/397 (37.3%) |
| Meditation app | 120/397 (30.2%) |
| Audio or video recording | 74/397 (18.6%) |
| Meditation class or course | 51/397 (12.8%) |
| Meditation retreat | 7/397 (1.8%) |
| Other | 31/397 (7.8%) |
| **Mindfulness training[b]** | |
| None | 243/310 (78.3%) |
| Mindfulness-Based Stress Reduction (MBSR) | 16/310 (5.2%) |
| Undergraduate or graduate coursework | 12/310 (3.9%) |
| Non-MBSR mindfulness class or course | 10/310 (3.2%) |
| At work or a professional event | 9/310 (2.9%) |
| In a yoga class | 5/310 (1.6%) |
| Meditation app | 4/310 (1.3%) |
| In psychotherapy | 4/310 (1.3%) |
| Other | 1/310 (3.5%) |
| **Meditation practice[c]** | |
| Meditate at least once a week | 20/241 (8.3%) |
| Days meditate per week (mean (SD)) | 5.1 (1.4) |
| Duration of meditation | |
| 1-9 min | 3/20 (15.0%) |
| 10-19 min. | 15/20 (75.0%) |
| 20-29 min | 2/20 (10.0%) |

[a]Asked at T0 without mention of mindfulness (to support blinding to which meditation arm was the intervention); check all that apply

[b]Asked at T1; check all that apply

[c]Participants with prior meditation training were asked if they meditate at least once a week and those who responded yes were asked how many days/week they meditate and how long they meditate for

medRxiv preprint doi: https://doi.org/10.64898/2026.01.14.26344130; this version posted January 16, 2026. The copyright holder has placed this preprint (which was not certified by peer review) in the Public Domain. It is no longer restricted by copyright. Anyone can legally share, reuse, remix, or adapt this material for any purpose without crediting the original authors.

## Supplemental Table 4. Meditation Adherence

| Adherence Metric | MM (n=132) | ACM (n=133) | p-value |
|---|---|---|---|
| Any meditation sessions, n (%) | 106 (80.3%) | 88 (66.2%) | 0.009 |
| Meditation dose (all participants)[a] | 13.0 (30.8) | 5.0 (16.0) | 0.0001 |
| Meditation dose (among those who meditated)[a] | 17.0 (30.8) | 12.0 (17.2) | 0.004 |

[a] Median number of sessions initiated (IQR)

1. Abbreviations:
   - MM: Mindfulness Meditation
   - ACM: Active Control Meditation
   - NMC: No-Meditation Control
   - IQR: Inter-Quartile Range

## Supplemental Table 5. Adherence to Meditation Instructions

| Instruction Type | MM (n=100) | ACM (n=90) | p-value |
|---|---|---|---|
| Mindfulness techniques score[a] (mean (SD)) | 14.9 (4.2) | 9.0 (3.8) | <0.001 |
| Active control techniques score[a] (mean (SD)) | 10.1 (3.9) | 14.8 (4.1) | <0.001 |
| Self-reported mindfulness techniques[b] (n (%)) | 63/77 (81.8%) | 7/62 (11.3%) | <0.001 |
| Self-reported ACM techniques[#] (n (%)) | 2/77 (2.6%) | 43/62 (69.4%) | <0.001 |

[a] Scores range from 0-20, with higher scores indicating better adherence to the respective meditation type's specific instructions based on Likert-scale responses to 5 questions per meditation type

[b] Based on quantitative content analysis of open-ended responses describing meditation practice; participants were coded as using either MM or ACM techniques if they mentioned at least one technique specific to that meditation type

1. Abbreviations:
   - MM: Mindfulness Meditation
   - ACM: Active Control Meditation
   - NMC: No-Meditation Control
   - SD: Standard Deviation

**Supplemental Figure 1: Participant descriptions of what they did while meditating**

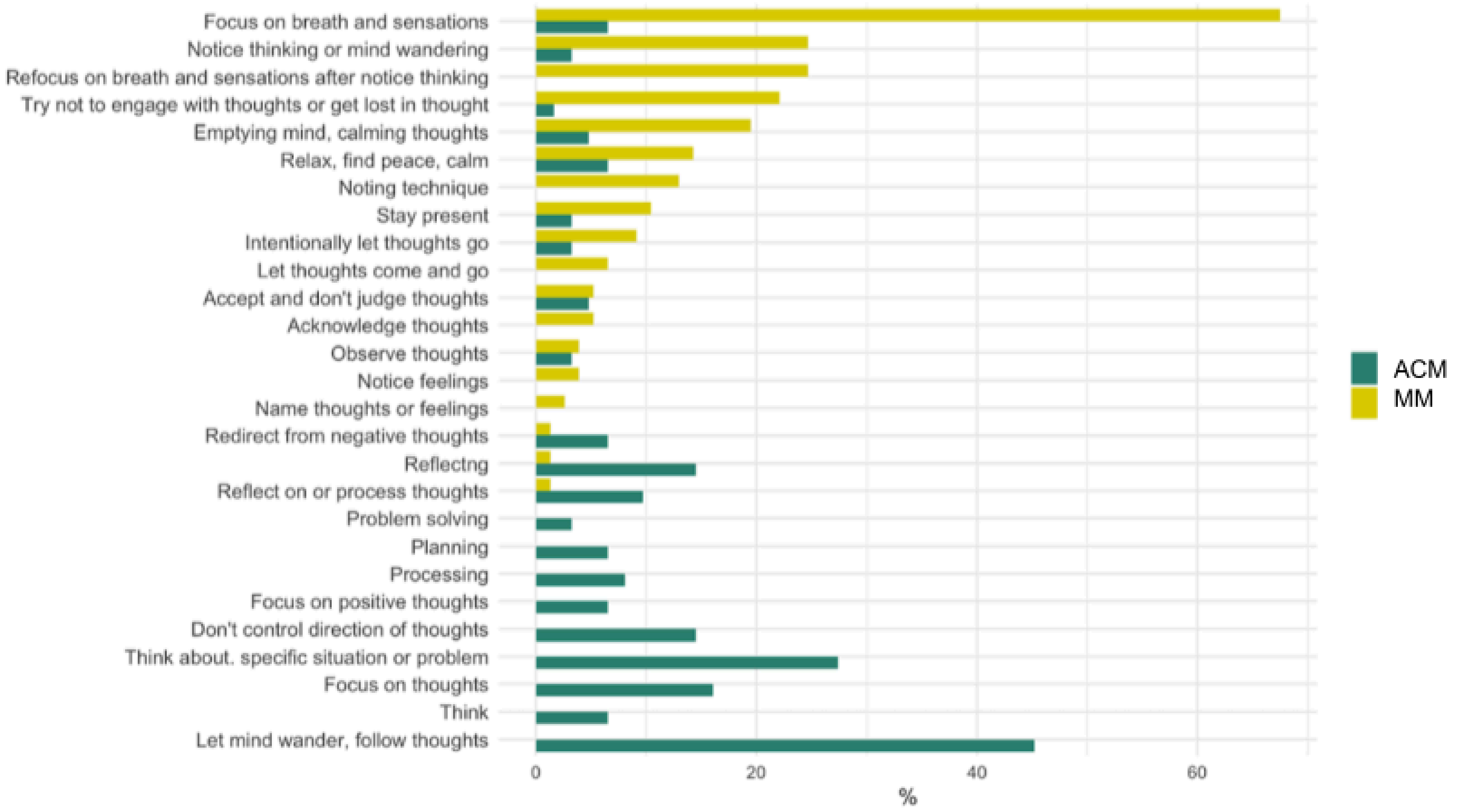


Frequency of meditation techniques reported by participants in open-ended responses describing their meditation practice during the final week of the intervention. Yellow bars represent mindfulness meditation (MM) participants (n=77); teal bars represent active control meditation (ACM) participants (n=62). Data analyzed using quantitative content analysis and inductive coding demonstrate that participants in each arm engaged in fundamentally different practices consistent with their assigned meditation type, suggesting participants understood and followed the meditation instructions they were randomized to.

**Supplemental Table 6. Participant-reported harms and negative effects**

| | All[a] | MM | ACM | p value[b] | Examples |
|---|---|---|---|---|---|
| **Difficulty fitting meditation in** | 9/189 (4.8%) | 5/99 (5.1%) | 4/90 (4.4%) | 0.7 | I didn't always feel good about starting to meditate- sometimes I felt like I couldn't afford the time<br><br>I found it really hard to establish the routine of meditating every day. |
| **Stress, anxiety about having to meditate** | 11/189 (5.8%) | 6/99 (6.1%) | 5/90 (5.6%) | 1.0 | I found the requirement to meditate stressful<br><br>It did sometimes feel like another thing to have to do and caused a little stress on the front end at times |
| **Guilt, stress because didn't meditate or perceived didn't meditate well enough** | 12/189 (6.3%) | 6/99 (6.1%) | 6/90 (6.7%) | 1.0 | I definitely felt bad and was hard on myself whenever I missed days<br><br>Stress of getting the reminders and feeling like I was failing the study when I didn't get to my daily meditation. |
| **Difficulty with specific type of meditation** | 19/189 (10.5%) | 1/99 (1.0%) | 18/90 (20.0%) | 0.001 | I often meditated before bed as that's when I had the time, focusing on my thoughts at that time sometimes made it harder to go to sleep. |

| | | | | | |
|---|---|---|---|---|---|
| | | | | | I don't know that I will continue the type I was assigned. Getting absorbed in my thoughts was not as helpful to me |
| **Increased distress due to meditation session** | 6/189 (3.2%) | 1/99 (1.0%) | 5/90 (5.6%) | 0.1 | About halfway through I felt that reflecting on my thoughts seemed to exacerbate the stress that I was experiencing in my day to day life.<br>The reflective meditation often left me feeling agitated when it was over. |
| **Increased anxiety due to greater awareness** | 1/189 (0.5%) | 1/99 (1.0%) | 0/90 (0.0%) | 1.0 | Sometimes being aware of myself made me incredibly anxious. Like life was "too real".<br>I think the meditation has made me more aware of my emotions throughout the day, so I may be experiencing more moments of anxiety |

[a]189 participants in the MM and ACM arms completed the T1 survey, which included the questions these data arise from

[b]Fisher's exact test

MM = mindfulness meditation arm; ACM = active control meditation arm

medRxiv preprint doi: https://doi.org/10.64898/2026.01.14.26344130; this version posted January 16, 2026. The copyright holder has placed this preprint (which was not certified by peer review) in the Public Domain. It is no longer restricted by copyright. Anyone can legally share, reuse, remix, or adapt this material for any purpose without crediting the original authors.

**Supplemental Table 7. Five-Factor Mindfulness Questionnaire Subscale Scores by Timepoint and Study Arm**

| Arm | Subscale | T0 | T1 | p-value |
|---|---|---|---|---|
| ACM | Observing | 23.9 | 27.4 | <0.0001 |
| | Describing | 27.7 | 29.3 | 0.0007 |
| | Acting with awareness | 23.6 | 26.1 | 0.0001 |
| | Non-reactivity of inner experience | 19.8 | 22.7 | <0.0001 |
| | Non-judging of inner experience | 25.3 | 28.9 | <0.0001 |
| NMC | Observing | 24.0 | 24.5 | 0.085 |
| | Describing | 27.2 | 27.2 | 0.36 |
| | Acting with awareness | 24.2 | 23.8 | 0.35 |
| | Non-reactivity of inner experience | 19.5 | 20.1 | 0.026 |
| | Non-judging of inner experience | 24.9 | 25.2 | 0.97 |
| MM | Observing | 23.9 | 27.2 | <0.0001 |
| | Describing | 26.9 | 29.1 | 0.0001 |
| | Acting with awareness | 23.5 | 25.3 | 0.017 |
| | Non-reactivity of inner experience | 19.5 | 22.0 | <0.0001 |
| | Non-judging of inner experience | 25.8 | 28.6 | 0.0003 |

Abbreviations: ACM, Active control Meditation; MM, Mindfulness Meditation; NMC, No-Meditation Control

medRxiv preprint doi: https://doi.org/10.64898/2026.01.14.26344130; this version posted January 16, 2026. The copyright holder has placed this preprint (which was not certified by peer review) in the Public Domain. It is no longer restricted by copyright. Anyone can legally share, reuse, remix, or adapt this material for any purpose without crediting the original authors.

**Supplemental Table 8. Linear Regression Results for Intention-to-Treat Outcome Analyses for Burnout subscales**

| Outcome | Comparison | Term | Coefficient (β) | Standard Error | p-value |
|---|---|---|---|---|---|
| Work Exhaustion | MM vs ACM | Study Arm | -0.32 | 0.37 | 0.40 |
| | | T0 Work Exhaustion | 0.29 | 0.06 | < 0.001 |
| | | T0 Mindfulness | -0.01 | 0.05 | 0.88 |
| | ACM vs NMC | Study Arm | 1.49 | 0.38 | < 0.001 |
| | | T0 Work Exhaustion | 0.46 | 0.06 | < 0.001 |
| | | T0 Mindfulness | 0.01 | 0.05 | 0.87 |
| | MM vs NMC | Study Arm | -1.89 | 0.40 | < 0.001 |
| | | T0 Work Exhaustion | 0.43 | 0.06 | < 0.001 |
| | | T0 Mindfulness | -0.02 | 0.05 | 0.75 |
| Interpersonal Disengagement | MM vs ACM | Study Arm | -0.21 | 0.60 | 0.73 |
| | | T0 Interpersonal Disengagement | 0.26 | 0.07 | < 0.001 |
| | | T0 Mindfulness | 0.02 | 0.06 | 0.70 |
| | ACM vs NMC | Study Arm | 1.84 | 0.56 | 0.002 |
| | | T0 Interpersonal Disengagement | 0.45 | 0.06 | < 0.001 |
| | | T0 Mindfulness | -0.06 | 0.06 | 0.28 |
| | MM vs NMC | Study Arm | -2.07 | 0.56 | < 0.001 |
| | | T0 Interpersonal Disengagement | 0.36 | 0.06 | < 0.001 |
| | | T0 Mindfulness | -0.04 | 0.06 | 0.48 |

1. Abbreviations:
   - MM: Mindfulness Meditation
   - ACM: Active Control Meditation
   - NMC: No-Meditation Control
2. Sample Size: N = 397

medRxiv preprint doi: https://doi.org/10.64898/2026.01.14.26344130; this version posted January 16, 2026. The copyright holder has placed this preprint (which was not certified by peer review) in the Public Domain. It is no longer restricted by copyright. Anyone can legally share, reuse, remix, or adapt this material for any purpose without crediting the original authors.

**Supplemental Table 9. Linear Regression Results for Intention-to-Treat Outcome Analyses for Secondary Outcomes**

| Outcome | Comparison | Term | Coefficient (β) | Standard Error | p-value |
|---|---|---|---|---|---|
| **Stress** | MM vs ACM | Study Arm | 0.39 | 0.98 | 0.695 |
| | | T0 Stress | 0.6 | 0.11 | 0.0009 |
| | | T0 Mindfulness | -0.02 | 0.1 | 0.811 |
| | ACM vs NMC | Study Arm | 3.9 | 1.12 | 0.0066 |
| | | T0 Stress | 0.62 | 0.08 | <0.0001 |
| | | T0 Mindfulness | -0.03 | 0.09 | 0.772 |
| | MM vs NMC | Study Arm | -3.5 | 0.91 | 0.0012 |
| | | T0 Stress | 0.59 | 0.09 | 3e-05 |
| | | T0 Mindfulness | -0.04 | 0.1 | 0.688 |
| **Professional Fulfillment** | MM vs ACM | Study Arm | 0.91 | 0.68 | 0.187 |
| | | T0 Professional Fulfillment | 0.56 | 0.22 | 0.06 |
| | | T0 Mindfulness | 0.01 | 0.09 | 0.947 |
| | ACM vs NMC | Study Arm | -0.81 | 0.91 | 0.396 |
| | | T0 Professional Fulfillment | 0.68 | 0.18 | 0.014 |
| | | T0 Mindfulness | -0.07 | 0.07 | 0.388 |
| | MM vs NMC | Study Arm | 1.61 | 0.77 | 0.055 |
| | | T0 Professional Fulfillment | 0.53 | 0.14 | 0.01 |
| | | T0 Mindfulness | 0.03 | 0.08 | 0.698 |
| **Professional Self-Care** | MM vs ACM | Study Arm | 3.86 | 2.21 | 0.083 |
| | | T0 Professional Self-Care | 0.36 | 0.11 | 0.0056 |
| | | T0 Mindfulness | -0.13 | 0.28 | 0.639 |
| | ACM vs NMC | Study Arm | -2.78 | 2.59 | 0.295 |
| | | T0 Professional Self-Care | 0.57 | 0.09 | <0.0001 |
| | | T0 Mindfulness | -0.07 | 0.23 | 0.751 |
| | MM vs NMC | Study Arm | 6.54 | 2.27 | 0.0071 |
| | | T0 Professional Self-Care | 0.52 | 0.08 | <0.0001 |
| | | T0 Mindfulness | 0.11 | 0.22 | 0.618 |
| **Resilience** | MM vs ACM | Study Arm | 0.55 | 1.18 | 0.645 |
| | | T0 Resilience | 0.57 | 0.2 | 0.034 |
| | | T0 Mindfulness | -0.01 | 0.14 | 0.937 |

medRxiv preprint doi: https://doi.org/10.64898/2026.01.14.26344130; this version posted January 16, 2026. The copyright holder has placed this preprint (which was not certified by peer review) in the Public Domain. It is no longer restricted by copyright. Anyone can legally share, reuse, remix, or adapt this material for any purpose without crediting the original authors.

| | | | | | |
|---|---|---|---|---|---|
| | ACM vs NMC | Study Arm | -0.57 | 1.79 | 0.762 |
| | | T0 Resilience | 0.62 | 0.26 | 0.072 |
| | | T0 Mindfulness | 0.01 | 0.1 | 0.912 |
| | MM vs NMC | Study Arm | 1.15 | 1.18 | 0.352 |
| | | T0 Resilience | 0.59 | 0.14 | 0.0052 |
| | | T0 Mindfulness | -0.01 | 0.11 | 0.947 |
| **Reactive Distress** | MM vs ACM | Study Arm | -0.22 | 0.91 | 0.808 |
| | | T0 Reactive Distress | 0.61 | 0.1 | <0.0001 |
| | | T0 Mindfulness | -0.15 | 0.16 | 0.38 |
| | ACM vs NMC | Study Arm | 0.27 | 1.17 | 0.823 |
| | | T0 Reactive Distress | 0.65 | 0.11 | <0.0001 |
| | | T0 Mindfulness | -0.12 | 0.1 | 0.282 |
| | MM vs NMC | Study Arm | -0.49 | 1.14 | 0.678 |
| | | T0 Reactive Distress | 0.65 | 0.07 | <0.0001 |
| | | T0 Mindfulness | -0.05 | 0.11 | 0.677 |
| **Desire to Reduce Clinical Time** | MM vs ACM | Study Arm | 1.25 | 3.1 | 0.687 |
| | | T0 Desire to Reduce Clinical Time | 0.54 | 0.05 | <0.0001 |
| | | T0 Mindfulness | 0.5 | 0.32 | 0.122 |
| | ACM vs NMC | Study Arm | 3.67 | 2.96 | 0.219 |
| | | T0 Desire to Reduce Clinical Time | 0.59 | 0.05 | <0.0001 |
| | | T0 Mindfulness | 0.47 | 0.33 | 0.153 |
| | MM vs NMC | Study Arm | -2.67 | 2.89 | 0.357 |
| | | T0 Desire to Reduce Clinical Time | 0.59 | 0.05 | <0.0001 |
| | | T0 Mindfulness | 0.4 | 0.32 | 0.217 |

1. Abbreviations:
   - MM: Mindfulness Meditation
   - ACM: Active Control Meditation
   - NMC: No-Meditation Control
2. Sample Size: N = 397

medRxiv preprint doi: https://doi.org/10.64898/2026.01.14.26344130; this version posted January 16, 2026. The copyright holder has placed this preprint (which was not certified by peer review) in the Public Domain. It is no longer restricted by copyright. Anyone can legally share, reuse, remix, or adapt this material for any purpose without crediting the original authors.

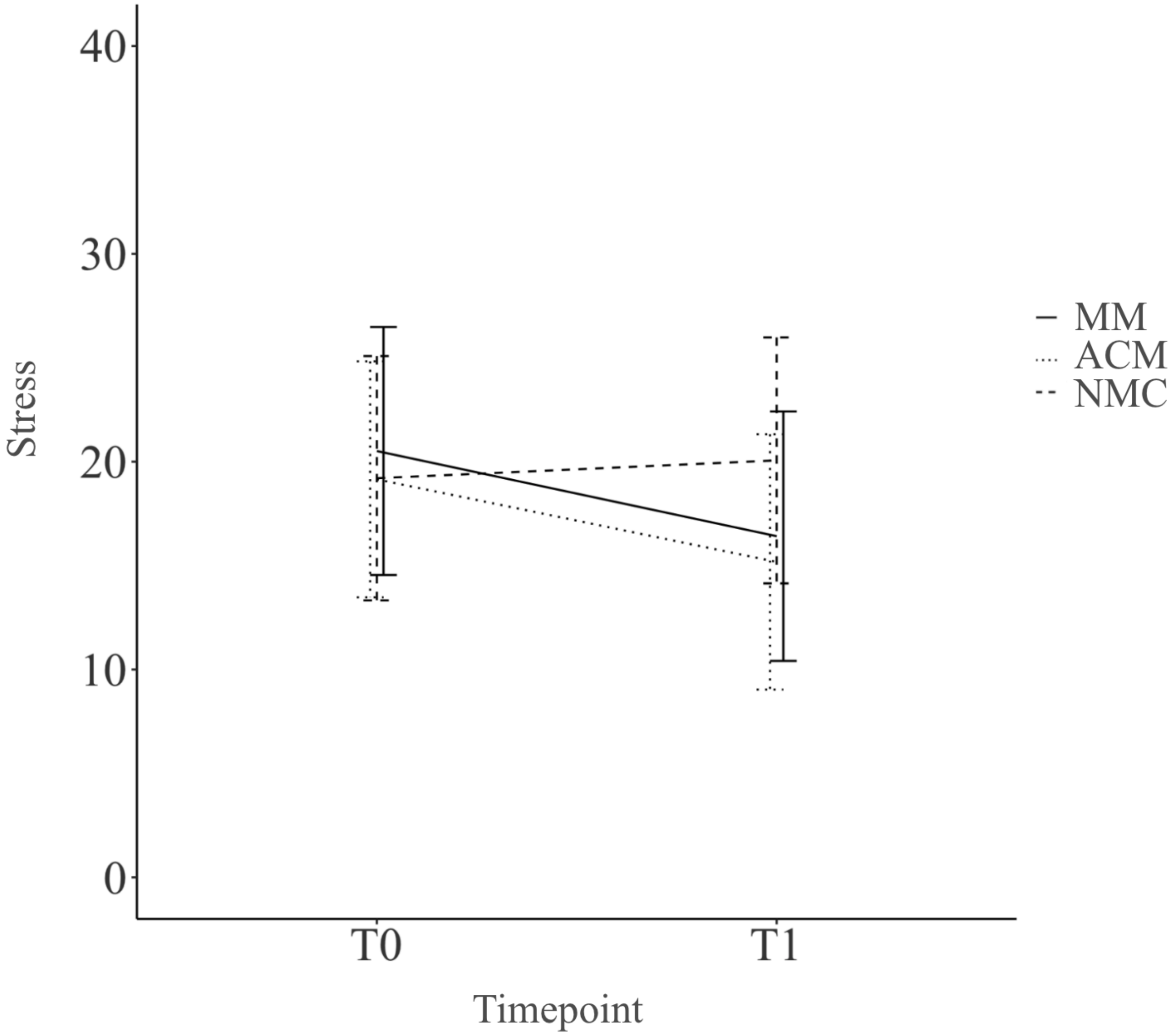


**Supplemental Figure 2. Stress by study arm and timepoint**

Mean perceived stress scores for each study arm at baseline (T0) and post-intervention (T1), measured using the Perceived Stress Scale. Error bars represent ± one standard deviation from the mean. Both mindfulness meditation (MM) and active control meditation (ACM) groups showed significant stress reduction compared to the no-meditation control (NMC) group, with no significant difference between meditation groups. Sample sizes: MM n=100, ACM n=90, NMC n=111 at T1.

medRxiv preprint doi: https://doi.org/10.64898/2026.01.14.26344130; this version posted January 16, 2026. The copyright holder has placed this preprint (which was not certified by peer review) in the Public Domain. It is no longer restricted by copyright. Anyone can legally share, reuse, remix, or adapt this material for any purpose without crediting the original authors.

**Supplemental Table 10: CONSORT 2025 checklist**

**Study Title:** The Effects of Mindfulness Meditation on Burnout in Clinical Genetic Counselors: A Three-Arm Randomized Controlled Trial

| Section/Topic | No | CONSORT 2025 checklist item description | Reported on page no. |
|---|---|---|---|
| **Title and abstract** | | | |
| Title and structured abstract | 1a | Identification as a randomized trial | 1 |
| | 1b | Structured summary of the trial design, methods, results, and conclusions | 2 |
| **Open science** | | | |
| Trial registration | 2 | Name of trial registry, identifying number (with URL) and date of registration | 1 |
| Protocol and statistical analysis plan | 3 | Where the trial protocol and statistical analysis plan can be accessed | 1,7 |
| Data sharing | 4 | Where and how the individual de-identified participant data (including data dictionary), statistical code and any other materials can be accessed | 1 |
| Funding and conflicts of interest | 5a | Sources of funding and other support (eg, supply of drugs), and role of funders in the design, conduct, analysis and reporting of the trial | 1-2 |
| | 5b | Financial and other conflicts of interest of the manuscript authors | 1 |
| **Introduction** | | | |
| Background and rationale | 6 | Scientific background and rationale | 3 |
| Objectives | 7 | Specific objectives related to benefits and harms | 3 |
| **Methods** | | | |
| Patient and public involvement | 8 | Details of patient or public involvement in the design, conduct and reporting of the trial | 7 |

medRxiv preprint doi: https://doi.org/10.64898/2026.01.14.26344130; this version posted January 16, 2026. The copyright holder has placed this preprint (which was not certified by peer review) in the Public Domain. It is no longer restricted by copyright. Anyone can legally share, reuse, remix, or adapt this material for any purpose without crediting the original authors.

| | | | |
|---|---|---|---|
| Trial design | 9 | Description of trial design including type of trial (eg, parallel group, crossover), allocation ratio, and framework (eg, superiority, equivalence, non-inferiority, exploratory) | 3 |
| Changes to trial protocol | 10 | Important changes to the trial after it commenced including any outcomes or analyses that were not prespecified, with reason | 4 |
| Trial setting | 11 | Settings (eg, community, hospital) and locations (eg, countries, sites) where the trial was conducted | 4 |
| Eligibility criteria | 12a | Eligibility criteria for participants | 5 |
| | 12b | If applicable, eligibility criteria for sites and for individuals delivering the interventions (eg, surgeons, physiotherapists) | N/A |
| Intervention and comparator | 13 | Intervention and comparator with sufficient details to allow replication. If relevant, where additional materials describing the intervention and comparator (eg, intervention manual) can be accessed | 4 |
| Outcomes | 14 | Prespecified primary and secondary outcomes, including the specific measurement variable (eg, systolic blood pressure), analysis metric (eg, change from baseline, final value, time to event), method of aggregation (eg, median, proportion), and time point for each outcome | 6 |
| Harms | 15 | How harms were defined and assessed (eg, systematically, non-systematically) | 7 |
| Sample size | 16a | How sample size was determined, including all assumptions supporting the sample size calculation | 4 |
| | 16b | Explanation of any interim analyses and stopping guidelines | N/A |
| **randomization:** | | | |
| Sequence generation | 17a | Who generated the random allocation sequence and the method used | 5 |
| | 17b | Type of randomization and details of any restriction (eg, stratification, blocking and block size) | 5 |

medRxiv preprint doi: https://doi.org/10.64898/2026.01.14.26344130; this version posted January 16, 2026. The copyright holder has placed this preprint (which was not certified by peer review) in the Public Domain. It is no longer restricted by copyright. Anyone can legally share, reuse, remix, or adapt this material for any purpose without crediting the original authors.

| | | | |
|---|---|---|---|
| Allocation concealment mechanism | 18 | Mechanism used to implement the random allocation sequence (eg, central computer/telephone; sequentially numbered, opaque, sealed containers), describing any steps to conceal the sequence until interventions were assigned | 5 |
| Implementation | 19 | Whether the personnel who enrolled and those who assigned participants to the interventions had access to the random allocation sequence | 5 |
| Blinding | 20a | Who was blinded after assignment to interventions (eg, participants, care providers, outcome assessors, data analysts) | 3-4 |
| | 20b | If blinded, how blinding was achieved and description of the similarity of interventions | 4 |
| Statistical methods | 21a | Statistical methods used to compare groups for primary and secondary outcomes, including harms | 7 |
| | 21b | Definition of who is included in each analysis (eg, all randomized participants), and in which group | 7 |
| | 21c | How missing data were handled in the analysis | 7 |
| | 21d | Methods for any additional analyses (eg, subgroup and sensitivity analyses), distinguishing prespecified from post hoc | 7 |
| **Results** | | | |
| Participant flow, including flow diagram | 22a | For each group, the numbers of participants who were randomly assigned, received intended intervention, and were analysed for the primary outcome | 14 |
| | 22b | For each group, losses and exclusions after randomization, together with reasons | 14 |
| Recruitment | 23a | Dates defining the periods of recruitment and follow-up for outcomes of benefits and harms | 5, 7 |
| | 23b | If relevant, why the trial ended or was stopped | N/A |
| Intervention and comparator delivery | 24a | Intervention and comparator as they were actually administered (eg, where appropriate, who delivered the intervention/comparator, how participants adhered, whether they were delivered as intended (fidelity)) | 7-8 |
| | 24b | Concomitant care received during the trial for each group | 8 |

medRxiv preprint doi: https://doi.org/10.64898/2026.01.14.26344130; this version posted January 16, 2026. The copyright holder has placed this preprint (which was not certified by peer review) in the Public Domain. It is no longer restricted by copyright. Anyone can legally share, reuse, remix, or adapt this material for any purpose without crediting the original authors.

| | | | |
|---|---|---|---|
| Baseline data | 25 | A table showing baseline demographic and clinical characteristics for each group | 15 |
| Numbers analysed, outcomes and estimation | 26 | For each primary and secondary outcome, by group: ● the number of participants included in the analysis ● the number of participants with available data at the outcome time point ● result for each group, and the estimated effect size and its precision (such as 95% confidence interval) ● for binary outcomes, presentation of both absolute and relative effect size | 8-9, 16 |
| Harms | 27 | All harms or unintended events in each group | 8, 24-25 |
| Ancillary analyses | 28 | Any other analyses performed, including subgroup and sensitivity analyses, distinguishing pre-specified from post hoc | 7-9 |
| **Discussion** | | | |
| Interpretation | 29 | Interpretation consistent with results, balancing benefits and harms, and considering other relevant evidence | 9-12 |
| Limitations | 30 | Trial limitations, addressing sources of potential bias, imprecision, generalisability, and, if relevant, multiplicity of analyses | 12 |
